# Supplementary material for: The Phase Diagram of the API Benzocaine and Its Highly Persistent, Metastable Crystalline Polymorphs
Source: Pharmaceutics. 2023 May 20;15(5):1549. doi: 10.3390/pharmaceutics15051549 (PMC10220575; doi:10.3390/pharmaceutics15051549)
Supplement: Supplementary file 1 [file pharmaceutics-15-01549-s001.zip › pharmaceutics-2359686-supplementary.pdf]

SUPPLEMENTARY MATERIALS TO:

The phase diagram of the API Benzocaine and its highly persistent, metastable crystalline polymorphs

Ivo B. Rietveld, Hiroshi Akiba, Osamu Yamamuro, Maria Barrio, René Céolin, Josep-Lluis Tamarit

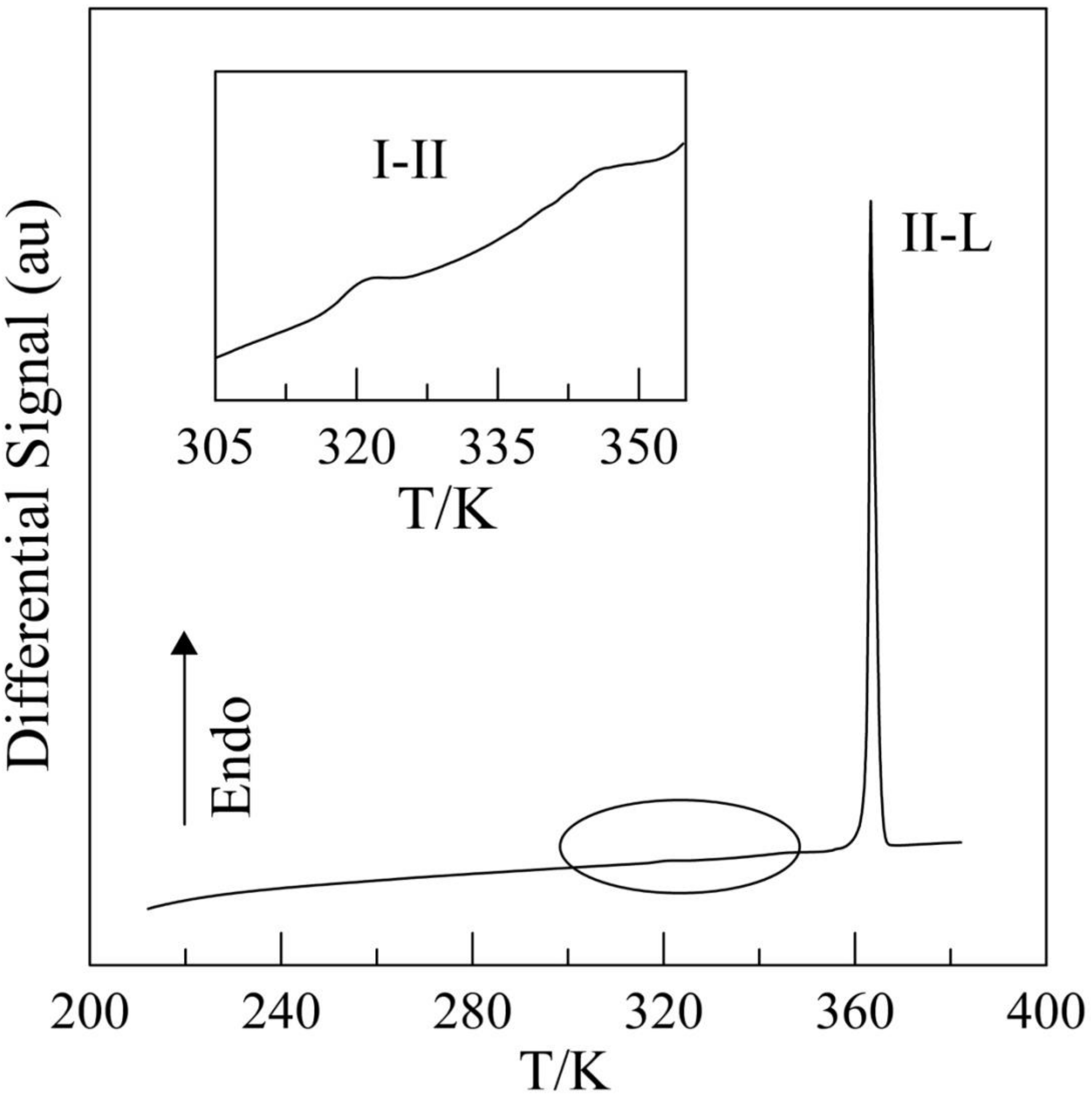

Figure S1. An example of the appearance of the I-II transition in a DSC measurement. Form I loaded into the capsule often leads to two small peaks after which the melting peak of form II is observed.

Table S1. Unit-cell parameters of form I ($P2_1/c$, Z = 4) as a function of temperature [a]

T /K	*a*(σ) /Å	*b*(σ) /Å	*c*(σ) /Å	*β* (σ) /°	*V*(σ) /Å^3	*V/Z* /Å^3	*v* /cm^{3}g^{-1}
**120**	8.2001(4)	5.4400(4)	19.6206(12)	91.297(4)	874.90(9)	218.72	0.79736
**170**	8.2130(5)	5.4509(4)	19.6913(10)	91.449(4)	881.26(9)	220.31	0.80315
**200**	8.2243(6)	5.4641(4)	19.7493(12)	91.555(6)	887.17(11)	221.79	0.80855
**225**	8.2253(5)	5.4711(4)	19.7913(10)	91.594(5)	890.29(10)	222.57	0.81139
**250**	8.2419(5)	5.4839(4)	19.8494(11)	91.658(4)	896.77(10)	224.19	0.81729
**275**	8.2483(5)	5.4935(4)	19.9059(11)	91.696(4)	901.57(9)	225.39	0.82167
**293**	8.2562(5)	5.5028(3)	19.9572(10)	91.743(4)	906.27(9)	226.57	0.82595
**310**	8.2594(6)	5.5094(4)	19.9962(12)	91.745(5)	909.49(11)	227.37	0.82889
**320**	8.2638(5)	5.5155(4)	20.0254(12)	91.729(5)	912.32(11)	228.08	0.83147

[a] (σ) standard deviation in the last digit

Table S2. Unit-cell parameters of form II ($P2_12_12_1$, Z = 4) as a function of temperature [a]

T /K	*a*(σ) /Å	*b*(σ) /Å	*c*(σ) /Å	*V*(σ) /Å^3	*V/Z* /Å^3	*v* /cm^{3}g^{-1}
**250**	8.2255(10)	5.2848(6)	20.8098(22)	904.61(18)	226.15	0.82444
**260**	8.2237(10)	5.283(6)	20.8289(21)	906.00(17)	226.50	0.82571
**270**	8.2306(8)	5.2964(5)	20.8431(19)	908.61(15)	227.15	0.82809
**280**	8.2332(9)	5.3015(5)	20.855(2)	910.28(16)	227.57	0.82961
**290**	8.2375(8)	5.3074(6)	20.8703(18)	912.43(15)	228.11	0.83156
**300**	8.2412(12)	5.3128(5)	20.888(3)	914.6(2)	228.64	0.83350
**320**	8.2497(10)	5.3244(6)	20.933(2)	919.46(18)	229.87	0.83797
**330**	8.2530(10)	5.3306(6)	20.961(2)	922.14(18)	230.53	0.84041
**335**	8.2501(15)	5.3315(10)	20.936(4)	920.9(3)	230.22	0.83928
**340**	8.2570(15)	5.3373(9)	20.966(3)	924.0(3)	230.99	0.84209
**345**	8.2671(6)	5.3464(4)	21.0205(12)	929.08(11)	232.27	0.84674
**350**	8.2610(10)	5.3427(6)	21.018(21)	927.64(18)	231.91	0.84543
**355**	8.2639(9)	5.3448(6)	21.040(2)	929.32(17)	232.33	0.84696

[a] (σ) standard deviation in the last digit(s)

Table S3. Unit-cell parameters of form III ($P2_1$, Z = 8) as a function of temperature [a]

T /K	*a*(σ) /Å	*b*(σ) /Å	*c*(σ) /Å	*γ* (σ) /°	*V*(σ) /Å³	*V/Z* /Å³	*v* /cm³g⁻¹
**120**	8.1908(9)	10.6294(11)	20.4674(19)	99.349(9)	1758.3(3)	219.79	0.80123
**170**	8.2021(10)	10.6653(11)	20.5263(19)	99.404(9)	1771.5(4)	221.43	0.80723
**200**	8.2090(10)	10.6900(10)	20.5656(18)	99.416(10)	1780.4(3)	222.55	0.81130
**220**	8.2173(10)	10.7125(11)	20.602(2)	99.414(9)	1789.1(4)	223.64	0.81526
**230**	8.2212(10)	10.7202(11)	20.622(3)	99.412(10)	1793.0(4)	224.13	0.81706
**240**	8.2228(10)	10.7290(11)	20.639(3)	99.388(10)	1796.4(4)	224.55	0.81860
**250**	8.2323(10)	10.7445(11)	20.668(3)	99.402(9)	1803.5(4)	225.44	0.82185
**260**	8.2313(19)	10.747(2)	20.698(3)	99.447(15)	1806.2(6)	225.77	0.82306
**245**	8.2284(10)	10.7392(12)	20.654(3)	99.399(9)	1800.6(4)	225.08	0.82051
**255**	8.2302(12)	10.7493(13)	20.680(3)	99.413(10)	1804.9(4)	225.61	0.82246

[a] (σ) standard deviation in the last digit(s)

Table S4. Heat capacity of benzocaine forms I, II, and III as a function of temperature obtained by adiabatic calorimetry.[a] Transition peaks are marked with yellow background.

**Form I – form II (stable)**		**Form III (metastable) – form II - melt**	
**T /K**	Cp /Jmol⁻¹K⁻¹	**T /K**	Cp /Jmol⁻¹K⁻¹
**0**	0 [b]	**0**	0 [c]
**1.000**	0.274 [b]	**1.000**	0.285 [c]
**2.000**	0.556 [b]	**2.000**	0.577 [c]
**3.000**	0.851 [b]	**3.000**	0.884 [c]
**4.000**	1.167 [b]	**4.000**	1.213 [c]
**5.000**	1.510 [b]	**5.000**	1.571 [c]
**6.000**	1.887 [b]	**6.000**	1.966 [c]
**7.000**	2.306 [b]	**7.000**	2.404 [c]
**8.000**	2.774 [b]	**8.000**	2.894 [c]
**9.000**	3.296 [b]	**9.000**	3.441 [c]
**10.000**	3.880 [b]	**10.000**	4.055 [c]
**11.000**	4.533 [b]	**11.000**	4.741 [c]
**12.000**	5.262 [b]	**11.382**	4.747
**12.588**	5.656	**13.512**	6.848
**13.940**	6.770	**15.210**	8.749
**15.153**	8.142	**16.671**	10.581
**16.256**	9.534	**17.975**	12.279
**17.335**	10.907	**19.168**	13.755
**18.454**	12.279	**20.279**	15.392
**19.281**	13.304	**21.327**	16.734
**20.274**	14.595	**22.336**	18.022
**21.239**	15.833	**23.313**	19.336
**22.180**	17.063	**24.266**	20.730
**23.100**	18.353	**25.230**	21.990
**24.345**	20.356	**26.231**	23.219

**25.827**	22.352	**27.255**	24.409
**27.341**	24.167	**28.277**	25.652
**28.876**	26.485	**29.245**	27.405
**30.536**	28.701	**31.224**	29.330
**32.202**	30.525	**32.239**	30.692
**33.776**	32.760	**33.355**	32.257
**35.371**	34.752	**34.524**	33.851
**36.991**	36.714	**35.673**	35.300
**38.636**	38.810	**36.876**	36.741
**40.308**	40.883	**38.195**	38.352
**41.906**	42.762	**39.620**	40.110
**43.462**	44.561	**41.143**	41.950
**45.005**	46.206	**42.694**	43.772
**46.524**	47.900	**44.212**	45.401
**48.026**	49.468	**45.706**	47.001
**49.514**	51.158	**47.181**	48.603
**50.986**	52.706	**48.680**	50.230
**52.437**	54.260	**50.199**	51.908
**53.870**	55.640	**51.691**	53.123
**55.289**	57.021	**53.158**	55.164
**56.695**	58.060	**54.609**	56.204
**58.090**	60.128	**56.046**	57.622
**59.476**	60.805	**57.469**	59.005
**60.857**	62.227	**58.881**	60.309
**62.233**	63.559	**60.283**	61.617
**63.604**	64.795	**61.678**	62.912
**64.972**	66.083	**63.068**	63.951
**66.337**	67.371	**64.451**	65.861
**67.701**	68.516	**65.832**	66.622
**69.065**	69.658	**67.211**	67.104
**70.680**	70.975	**68.529**	69.104
**72.028**	72.067	**69.907**	70.406
**73.378**	72.873	**71.284**	71.227
**74.731**	74.676	**72.663**	72.423
**76.087**	75.296	**74.043**	73.480
**77.448**	76.377	**75.424**	74.530
**78.812**	76.644	**76.808**	75.670
**80.181**	78.252	**78.195**	76.818
**81.554**	79.527	**79.585**	77.754
**82.932**	80.274	**80.978**	78.831
**84.316**	81.611	**82.377**	79.958
**85.705**	83.011	**83.779**	80.981
**87.100**	83.825	**85.187**	82.113

**88.504**	84.646	**86.599**	83.044
**90.077**	86.099	**87.600**	83.828
**91.654**	87.202	**89.161**	85.089
**93.234**	88.252	**90.725**	86.081
**94.819**	89.437	**92.292**	87.543
**96.407**	90.639	**93.863**	88.555
**98.001**	91.844	**95.439**	89.707
**99.600**	92.975	**97.020**	90.846
**101.205**	94.338	**98.606**	92.027
**102.815**	95.367	**100.198**	92.976
**104.432**	96.535	**101.796**	94.339
**106.055**	97.617	**103.399**	95.369
**107.686**	98.746	**105.010**	96.650
**109.323**	99.910	**106.628**	97.799
**110.968**	101.234	**108.252**	98.996
**112.621**	102.190	**109.885**	100.247
**114.281**	103.421	**111.525**	101.212
**115.951**	104.256	**113.172**	102.323
**117.628**	105.528	**114.829**	103.455
**119.314**	106.809	**116.493**	104.482
**121.008**	107.623	**118.166**	105.601
**122.712**	108.910	**119.848**	106.897
**124.425**	110.003	**121.539**	107.821
**126.147**	111.178	**123.238**	109.022
**127.879**	112.335	**124.947**	110.102
**129.620**	113.338	**126.666**	111.302
**131.371**	114.208	**128.394**	112.438
**133.132**	115.714	**130.132**	113.526
**134.902**	117.052	**131.880**	115.000
**136.683**	118.219	**133.637**	115.956
**138.474**	118.707	**135.405**	117.075
**140.275**	120.343	**137.182**	118.265
**142.087**	121.613	**138.971**	119.158
**143.909**	122.912	**140.770**	120.541
**145.741**	124.434	**142.579**	121.740
**147.586**	125.335	**144.398**	123.011
**149.439**	126.982	**146.229**	124.283
**151.304**	127.530	**148.070**	125.831
**153.180**	128.682	**149.922**	126.466
**155.066**	130.424	**151.785**	127.846
**156.965**	131.287	**153.659**	128.890
**158.874**	132.606	**155.544**	129.832
**160.794**	133.631	**157.440**	131.084

**162.726**	134.875	**159.280**	132.767
**164.669**	136.019	**161.199**	133.705
**166.624**	137.874	**163.129**	134.720
**168.590**	138.878	**165.070**	136.180
**170.567**	140.138	**167.024**	136.503
**172.555**	141.925	**168.982**	138.527
**174.556**	143.043	**170.947**	140.028
**176.577**	144.191	**172.933**	141.278
**178.624**	145.208	**174.939**	142.381
**180.690**	147.046	**176.965**	143.623
**182.777**	147.561	**179.011**	145.058
**184.883**	149.615	**181.077**	146.240
**187.008**	151.038	**183.162**	147.522
**189.153**	152.160	**185.267**	149.349
**191.317**	153.694	**187.392**	149.584
**193.503**	155.046	**189.537**	151.756
**195.705**	156.620	**191.700**	153.108
**197.928**	158.142	**193.883**	154.713
**200.169**	159.873	**196.086**	156.118
**202.432**	160.958	**198.308**	157.479
**204.712**	162.960	**200.549**	159.306
**207.011**	164.760	**202.810**	160.905
**209.330**	165.289	**205.090**	161.248
**211.669**	168.344	**207.391**	164.085
**214.025**	169.577	**209.708**	165.366
**216.401**	171.843	**212.046**	166.999
**218.797**	173.051	**214.402**	168.727
**221.210**	174.634	**216.777**	170.532
**223.642**	176.325	**219.172**	171.619
**226.094**	176.777	**221.587**	174.468
**228.564**	180.441	**224.019**	175.482
**231.052**	182.043	**226.469**	177.238
**233.558**	184.642	**228.939**	179.002
**236.085**	184.439	**231.426**	181.817
**238.629**	187.259	**233.936**	182.946
**241.190**	189.245	**236.461**	184.675
**243.770**	192.210	**239.005**	186.341
**246.372**	193.224	**241.567**	188.367
**248.988**	195.119	**244.148**	190.231
**251.623**	196.947	**246.746**	193.533
**254.277**	196.949	**249.367**	193.623
**256.950**	201.468	**251.669**	195.901
**259.638**	203.188	**253.651**	197.227

**262.345**	204.906	**255.641**	198.931
**265.070**	207.168	**257.641**	200.612
**267.814**	207.126	**259.648**	202.643
**270.576**	212.165	**261.663**	206.527
**273.352**	215.573	**263.681**	218.256
**276.147**	216.279	**265.695**	238.330
**278.960**	218.560	**267.664**	353.318
**281.791**	220.905	**269.632**	251.630
**284.638**	225.065	**271.673**	213.196
**287.508**	224.720	**273.740**	215.803
**290.391**	228.455	**275.815**	215.157
**291.471**	230.283	**278.731**	221.728
**294.408**	232.735	**280.841**	220.199
**297.396**	235.423	**282.967**	223.502
**300.413**	234.870	**285.113**	225.344
**303.457**	241.403	**287.278**	227.454
**306.518**	244.248	**289.400**	229.619
**309.601**	253.014	**292.211**	232.245
**312.702**	264.798	**295.047**	235.225
**315.816**	276.471	**297.909**	237.553
**318.915**	333.731	**300.796**	238.982
**321.998**	334.508	**303.708**	242.781
**325.127**	276.204	**306.645**	246.056
**328.308**	266.738	**309.605**	249.168
**331.500**	271.201	**312.590**	252.397
**334.699**	274.121	**315.599**	255.699
**337.907**	278.116	**318.633**	258.480
**341.122**	282.803	**321.691**	261.323
**344.342**	290.261	**324.772**	264.222
		**327.878**	267.234
		**331.400**	270.478
		**334.214**	273.477
		**337.037**	276.667
		**339.869**	280.254
		**342.708**	285.663
		**345.552**	294.049
		**348.396**	307.854
		**351.230**	335.052
		**354.026**	398.251
		**356.714**	576.973
		**359.084**	1228.500
		**360.753**	3957.290
		**361.611**	12550.000

		**362.028**	21454.200
		**363.391**	892.051
		**366.057**	336.660
		**368.989**	336.885

[a] Uncertainty in last digit
[b] Data obtained by extrapolation with equation S1.
[c] Data obtained by extrapolation with equation S2.

EXTRAPOLATION TO 0K

Form I

$C_{p,I} = 0.2732\ T + 1.147\times10^{-3}\ T^3$ $R^2 = 0.997$ (S1)

Form II

$C_{p,III} = 0.2837\ T + 1.217\times10^{-3}\ T^3$ $R^2 = 0.996$ (S2)

Table S5. Temperature-pressure data of the I-II, I-*l*, and II-*l* equilibria

Transition I-II		Fusion II-*l*		Fusion I-*l*	
T /K	P /MPa	T /K	P /MPa	T /K	P /Mpa
**332.15**	27.5	**363.15**	8.3	**384.15**	144.5
**334.15**	31.5	**367.15**	32.1	**388.15**	174.8
**338.15**	41.0	**368.15**	37.7	**391.15**	185.3
**341.65**	50.5	**369.15**	46.7	**396.15**	212.4
**344.15**	51.5	**370.15**	55.3	**398.15**	227.8
**351.15**	63.1	**373.15**	67.4	**400.15**	244.8
**358.15**	83.6	**374.15**	86.4		
**368.15**	95.6	**378.15**	109.9		
**376.15**	110.9	**378.15**	97.7		

Table S6. Entropy (S), Enthalpy (H), and Gibbs free energy (G) of the stable form I and form II as obtained from the heat capacity measurements (Table S1). Transition peaks are marked with a yellow background.

Form I – form II (stable)			
**Temperature /K**	Entropy /J K^{-1} mol^{-1}	Enthalpy /J mol^{-1}	Gibbs free energy /J mol^{-1}
**0**	0 [a]	0 [a]	0 [a]
**1.000**	0.274421 [a]	0.137211 [a]	-0.13721 [a]
**2.000**	0.551137 [a]	0.552285 [a]	-0.549989 [a]
**3.000**	0.832442 [a]	1.25555 [a]	-1.241776 [a]
**4.000**	1.12063 [a]	2.26421 [a]	-2.21831 [a]
**5.000**	1.418 [a]	3.60236 [a]	-3.48764 [a]

**6.000**	1.72684 [a]	5.30098 [a]	-5.06006 [a]
**7.000**	2.04945 [a]	7.39793 [a]	-6.94822 [a]
**8.000**	2.38812 [a]	9.93797 [a]	-9.16699 [a]
**9.000**	2.74515 [a]	12.9727 [a]	-11.73365 [a]
**10.000**	3.12283 [a]	16.5607 [a]	-14.6676 [a]
**11.000**	3.52345 [a]	20.7673 [a]	-17.99065 [a]
**12.000**	3.94932 [a]	25.6647 [a]	-21.72714 [a]
**12.588**	4.21037	28.874	-24.125717
**13.940**	4.84353	37.2722	-30.245155
**15.153**	5.46522	46.3154	-36.496893
**16.256**	6.0863	56.0691	-42.87101
**17.335**	6.74286	67.0963	-49.791852
**18.454**	7.46772	80.0674	-57.741158
**19.281**	8.02866	90.6509	-64.152102
**20.274**	8.72892	104.501	-72.47087
**21.239**	9.43614	119.18	-81.234177
**22.180**	10.1489	134.654	-90.446572
**23.100**	10.8688	150.952	-100.11945
**24.345**	11.8846	175.049	-114.28396
**25.827**	13.1459	206.691	-132.82816
**27.341**	14.4708	241.912	-153.73704
**28.877**	15.854	280.793	-177.01503
**30.536**	17.3953	326.579	-204.6004
**32.202**	18.9686	375.93	-234.90255
**33.776**	20.478	425.724	-265.93888
**35.371**	22.0351	479.56	-299.83912
**36.991**	23.6352	537.453	-336.83668
**38.636**	25.2777	599.562	-377.05963
**40.308**	26.9661	666.206	-420.74895
**41.906**	28.5914	733.014	-465.12863
**43.462**	30.1829	800.949	-510.84813
**45.005**	31.766	870.971	-558.64195
**46.524**	33.3282	942.467	-608.09418
**48.026**	34.8747	1015.57	-659.30839
**49.514**	36.4103	1090.47	-712.35688
**50.986**	37.9313	1166.9	-767.06147
**52.437**	39.4319	1244.5	-823.18265
**53.870**	40.9137	1323.26	-880.7692
**55.289**	42.3778	1403.17	-939.84347
**56.695**	43.823	1484.09	-1000.4594
**58.090**	45.2592	1566.51	-1062.5924
**59.476**	46.6848	1650.31	-1126.3058

**60.857**	48.0971	1735.29	-1191.76
**62.233**	49.5027	1821.8	-1258.8867
**63.604**	50.9014	1909.8	-1327.7276
**64.972**	52.2936	1999.3	-1398.2936
**66.337**	53.6814	2090.42	-1470.6484
**67.701**	55.0644	2183.1	-1544.8315
**69.065**	56.4421	2277.31	-1620.858
**70.680**	58.067	2390.85	-1713.2965
**72.028**	59.4183	2487.27	-1792.4935
**73.378**	60.7647	2585.16	-1873.6565
**74.731**	62.1122	2684.95	-1956.763
**76.087**	63.4608	2786.64	-2041.9146
**77.448**	64.8048	2889.82	-2129.1692
**78.813**	66.1412	2994.23	-2218.5233
**80.181**	67.4743	3100.21	-2309.9334
**81.554**	68.8141	3208.56	-2403.5189
**82.933**	70.1531	3318.68	-2499.292
**84.316**	71.4924	3430.68	-2597.2803
**85.705**	72.8373	3545.01	-2697.5254
**87.100**	74.1842	3661.38	-2800.0861
**88.504**	75.5307	3779.61	-2905.144
**90.077**	77.035	3913.93	-3025.1594
**91.654**	78.5387	4050.57	-3147.816
**93.234**	80.0385	4189.21	-3273.1315
**94.819**	81.5355	4329.97	-3401.1283
**96.408**	83.0316	4473.02	-3531.849
**98.001**	84.5276	4618.43	-3665.3762
**99.600**	86.023	4766.19	-3801.7094
**101.205**	87.5198	4916.46	-3940.9814
**102.815**	89.017	5069.19	-4083.0929
**104.432**	90.5141	5224.33	-4228.2385
**106.055**	92.0116	5381.93	-4376.3602
**107.686**	93.5094	5542	-4527.6532
**109.323**	95.0084	5704.65	-4681.9533
**110.968**	96.5105	5870.1	-4839.4772
**112.621**	98.014	6038.18	-5000.2547
**114.281**	99.519	6208.93	-5164.2008
**115.951**	101.025	6382.24	-5331.7098
**117.628**	102.531	6558.15	-5502.3665
**119.314**	104.042	6737.15	-5676.5172
**121.008**	105.554	6918.86	-5854.0184
**122.712**	107.068	7103.32	-6035.2084

**124.425**	108.585	7290.78	-6219.9086
**126.147**	110.105	7481.23	-6408.1854
**127.879**	111.628	7674.74	-6600.137
**129.620**	113.154	7871.22	-6795.8015
**131.371**	114.681	8070.43	-6995.3277
**133.132**	116.212	8272.86	-7198.676
**134.902**	117.749	8478.91	-7405.6656
**136.683**	119.292	8688.4	-7616.7884
**138.474**	120.834	8900.59	-7831.7773
**140.275**	122.379	9115.9	-8050.8142
**142.087**	123.931	9335.05	-8273.934
**143.909**	125.489	9557.83	-8501.1665
**145.741**	127.054	9784.45	-8732.527
**147.586**	128.624	10014.7	-8968.4017
**149.439**	130.199	10248.6	-9208.2084
**151.304**	131.777	10485.9	-9452.4872
**153.180**	133.355	10726.2	-9701.1189
**155.066**	134.941	10970.6	-9954.1611
**156.965**	136.534	11219.1	-10211.959
**158.874**	138.129	11471	-10474.107
**160.794**	139.728	11726.6	-10740.824
**162.726**	141.331	11985.9	-11012.328
**164.669**	142.939	12249.1	-11288.522
**166.624**	144.555	12516.8	-11569.532
**168.590**	146.178	12788.8	-11855.349
**170.567**	147.804	13064.6	-12145.885
**172.555**	149.439	13345	-12441.447
**174.557**	151.082	13630.2	-12742.221
**176.577**	152.735	13920.5	-13048.988
**178.624**	154.403	14216.6	-13363.481
**180.690**	156.083	14518.5	-13684.137
**182.777**	157.775	14826	-14011.641
**184.883**	159.477	15138.9	-14345.686
**187.008**	161.195	15458.3	-14686.455
**189.153**	162.924	15783.5	-15034.063
**191.317**	164.664	16114.4	-15388.622
**193.503**	166.417	16451.8	-15750.389
**195.705**	168.181	16795.1	-16118.763
**197.928**	169.958	17144.8	-16494.647
**200.169**	171.749	17501.2	-16877.626
**202.432**	173.552	17864.2	-17268.278
**204.712**	175.366	18233.5	-17666.025

**207.011**	177.196	18610.2	-18071.321
**209.330**	179.034	18992.9	-18484.287
**211.669**	180.888	19383.1	-18905.282
**214.025**	182.758	19781.2	-19333.581
**216.401**	184.642	20186.7	-19770.013
**218.797**	186.541	20600	-20214.611
**221.210**	188.448	21019.5	-20667.082
**223.642**	190.367	21446.2	-21127.857
**226.094**	192.292	21879	-21597.067
**228.564**	194.232	22320.2	-22074.243
**231.052**	196.195	22771.1	-22560.147
**233.558**	198.172	23230.6	-23054.056
**236.085**	200.159	23697	-23557.538
**238.629**	202.15	24169.8	-24069.052
**241.190**	204.16	24651.9	-24589.45
**243.770**	206.189	25144	-25118.693
**246.372**	208.235	25645.4	-25657.873
**248.988**	210.287	26153.4	-26205.54
**251.623**	212.35	26669.9	-26762.244
**254.277**	214.416	27192.6	-27328.457
**256.950**	216.5	27725.1	-27904.575
**259.638**	218.605	28269	-28489.165
**262.345**	220.722	28821.4	-29083.913
**265.070**	222.85	29382.7	-29688.15
**267.814**	224.984	29951.1	-30302.765
**270.576**	227.135	30530.2	-30927.08
**273.352**	229.318	31123.9	-31560.634
**276.147**	231.514	31727.3	-32204.597
**278.960**	233.718	32339	-32858.973
**281.791**	235.936	32961	-33523.641
**284.638**	238.178	33595.9	-34198.61
**287.508**	240.435	34241.4	-34885.586
**290.391**	242.695	34894.5	-35581.944
**292.949**	244.719	35485	-36205.186
**295.898**	247.05	36171.3	-36930.301
**298.899**	249.426	36877.9	-37675.282
**301.930**	251.795	37589.7	-38434.764
**304.983**	254.224	38326.9	-39207.098
**308.057**	256.674	39077.7	-39992.522
**311.149**	259.2	39859.9	-40789.921
**314.256**	261.832	40682.7	-41599.577
**317.376**	264.563	41545.3	-42420.647

**320.454**	267.783	42572.4	-43239.733
**323.542**	270.991	43605.3	-44071.67
**326.712**	273.685	44481.1	-44935.074
**329.905**	276.279	45332.7	-45813.123
**333.100**	278.893	46199.1	-46700.158
**336.303**	281.516	47077.2	-47597.475
**339.514**	284.159	47970.3	-48505.659
**342.732**	286.827	48880.4	-49424.391
**345.953**	289.542	49815.2	-50352.724

[a] Data obtained by extrapolation, stable form *H* and *G* set to 0.

Table S7. Entropy (S), Enthalpy (H), and Gibbs free energy (G) of the metastable form III and form II as obtained from the heat capacity measurements (Table S1). Transition peaks are marked with a yellow background.

**Form III (metastable) – form II (metastable, stable) – melt (stable)**			
**Temperature /K**	Entropy /J K^{-1} mol^{-1}	Enthalpy /J mol^{-1}	Gibbs free energy /J mol^{-1}
**0**	0.015 [a]	197.4 [a]	197.4 [a]
**1**	0.300052 [a]	197.542526 [a]	197.242474 [a]
**2**	0.587537 [a]	197.973754 [a]	196.79868 [a]
**3**	0.879889 [a]	198.70463 [a]	196.064963 [a]
**4**	1.17954 [a]	199.75341 [a]	195.03525 [a]
**5**	1.48892 [a]	201.14564 [a]	193.70104 [a]
**6**	1.81047 [a]	202.91416 [a]	192.05134 [a]
**7**	2.14662 [a]	205.09912 [a]	190.07278 [a]
**8**	2.4998 [a]	207.748 [a]	187.7496 [a]
**9**	2.87244 [a]	210.9155 [a]	185.06354 [a]
**10**	3.26699 [a]	214.6636 [a]	181.9937 [a]
**11**	3.68586 [a]	219.0618 [a]	178.51734 [a]
**11.3819**	3.84777	220.8737	177.078767
**13.5115**	4.83979	233.2211	167.828277
**15.2101**	5.7622	246.4677	158.824062
**16.6707**	6.64781	260.5847	149.761054
**17.9748**	7.50827	275.49	140.530348
**19.1681**	8.34465	291.023	131.071914
**20.2787**	9.16526	307.208	121.348442
**21.3266**	9.9744	324.04	111.319961
**22.3356**	10.7775	341.574	100.852071
**23.3134**	11.5777	359.837	89.9214488
**24.2656**	12.3795	378.912	78.5160048
**25.2296**	13.2116	399.503	66.1796166
**26.2313**	14.0916	422.147	52.5060129
**27.2547**	15.0029	446.518	37.6184614

**28.2766**	15.9242	472.097	21.8147663
**29.2449**	16.8173	497.783	5.96274323
**31.2241**	18.6743	553.929	-29.159211
**32.2395**	19.6346	584.402	-48.607687
**33.3552**	20.7053	619.517	-71.112423
**34.5241**	21.8437	658.156	-95.978083
**35.6734**	22.9759	697.895	-121.73347
**36.8756**	24.1696	741.196	-150.0725
**38.1945**	25.489	790.719	-182.82061
**39.6201**	26.9265	846.646	-220.18462
**41.1425**	28.4733	909.11	-262.35275
**42.6936**	30.0593	975.592	-307.74773
**44.2122**	31.6175	1043.299	-354.58023
**45.7058**	33.1524	1112.308	-402.94896
**47.1808**	34.6705	1182.812	-452.96993
**48.6805**	36.2167	1256.92	-506.12706
**50.199**	37.7853	1334.47	-562.31427
**51.6915**	39.3237	1412.85	-619.85104
**53.1583**	40.8386	1492.27	-678.64055
**54.6095**	42.3383	1573.08	-738.99339
**56.046**	43.8159	1654.83	-800.87593
**57.4689**	45.2779	1737.81	-864.26111
**58.8807**	46.7257	1822.03	-929.21192
**60.2831**	48.1606	1907.53	-995.74027
**61.6783**	49.5851	1994.4	-1063.9247
**63.0678**	50.9982	2082.53	-1133.8143
**64.4509**	52.4062	2172.31	-1205.3168
**65.8318**	53.8103	2263.78	-1278.6489
**67.2113**	55.197	2356.02	-1353.8421
**68.529**	56.5192	2445.76	-1427.4443
**69.907**	57.9079	2541.88	-1506.2876
**71.2838**	59.289	2639.38	-1586.9652
**72.6627**	60.6651	2738.42	-1669.67
**74.0427**	62.0375	2839.09	-1754.334
**75.4244**	63.4057	2941.34	-1840.9969
**76.8084**	64.7713	3045.28	-1929.6999
**78.1951**	66.1355	3151.01	-2020.462
**79.5853**	67.4975	3258.46	-2113.3488
**80.9784**	68.856	3367.52	-2208.3287
**82.3766**	70.2151	3478.53	-2305.5512
**83.7793**	71.5737	3591.4	-2404.9945
**85.1866**	72.9321	3706.17	-2506.6676

**86.5992**	74.2902	3822.82	-2610.6519
**87.5999**	75.2489	3906.32	-2685.4761
**89.1608**	76.7405	4038.15	-2804.0944
**90.7247**	78.2286	4171.99	-2925.2763
**92.2917**	79.7152	4308.03	-3049.0213
**93.8631**	81.2017	4446.39	-3175.4533
**95.4392**	82.6859	4586.86	-3304.6161
**97.02**	84.1689	4729.57	-3436.4967
**98.6061**	85.6516	4874.6	-3571.1702
**100.198**	87.133	5021.86	-3708.6923
**101.796**	88.6145	5171.48	-3849.1216
**103.399**	90.0973	5323.62	-3992.3507
**105.01**	91.5813	5478.26	-4138.6923
**106.628**	93.0675	5635.52	-4288.0814
**108.252**	94.5555	5795.39	-4440.432
**109.885**	96.0462	5957.98	-4596.0567
**111.525**	97.5386	6123.2	-4754.7924
**113.172**	99.0311	6290.88	-4916.6676
**114.829**	100.526	6461.27	-5082.0301
**116.493**	102.022	6634.33	-5250.5188
**118.166**	103.52	6810.05	-5422.4943
**119.848**	105.021	6988.74	-5597.8168
**121.539**	106.525	7170.28	-5776.662
**123.238**	108.031	7354.55	-5958.9744
**124.947**	109.54	7541.81	-6144.8844
**126.666**	111.052	7732.04	-6334.4726
**128.394**	112.568	7925.36	-6527.6958
**130.132**	114.087	8121.69	-6724.6795
**131.88**	115.611	8321.42	-6925.3587
**133.637**	117.14	8524.36	-7129.8782
**135.405**	118.671	8730.32	-7338.3268
**137.182**	120.206	8939.52	-7550.5795
**138.971**	121.744	9151.81	-7767.0754
**140.77**	123.285	9367.41	-7987.4195
**142.579**	124.832	9586.57	-8211.8517
**144.398**	126.384	9809.24	-8440.3568
**146.229**	127.941	10035.56	-8673.1245
**148.07**	129.506	10265.8	-8910.1534
**149.922**	131.074	10499.4	-9151.4762
**151.785**	132.644	10736.3	-9397.0695
**153.659**	134.219	10976.9	-9647.0573
**155.544**	135.796	11220.7	-9901.553

**157.44**	137.377	11468.1	-10160.535
**159.28**	138.91	11710.8	-10414.785
**161.199**	140.506	11966.5	-10682.927
**163.129**	142.103	12225.5	-10955.62
**165.07**	143.706	12488.5	-11233.049
**167.024**	145.31	12754.9	-11515.357
**168.982**	146.912	13024.1	-11801.384
**170.947**	148.523	13297.8	-12091.761
**172.933**	150.147	13577.2	-12388.171
**174.939**	151.783	13861.7	-12691.066
**176.965**	153.43	14151.4	-13000.34
**179.011**	155.089	14446.7	-13315.937
**181.077**	156.76	14747.6	-13638.031
**183.162**	158.441	15053.8	-13966.57
**185.267**	160.138	15366.3	-14301.987
**187.392**	161.843	15684	-14644.083
**189.537**	163.557	16007.1	-14993.003
**191.7**	165.287	16336.8	-15348.718
**193.883**	167.03	16672.8	-15711.477
**196.086**	168.785	17015.1	-16081.276
**198.308**	170.552	17363.5	-16458.326
**200.549**	172.332	17718.6	-16842.41
**202.81**	174.127	18080.5	-17234.197
**205.09**	175.928	18447.9	-17633.174
**207.391**	177.743	18822.1	-18040.199
**209.708**	179.573	19203.8	-18454.095
**212.046**	181.415	19592.2	-18876.125
**214.402**	183.27	19987.8	-19305.655
**216.777**	185.139	20390.7	-19743.177
**219.172**	187.019	20800.4	-20188.928
**221.587**	188.915	21218.3	-20642.808
**224.019**	190.825	21643.8	-21104.626
**226.469**	192.743	22075.9	-21574.414
**228.939**	194.675	22515.8	-22052.9
**231.426**	196.625	22964.6	-22539.537
**233.936**	198.592	23422.3	-23035.518
**236.461**	200.565	23886.5	-23539.3
**239.005**	202.55	24358.3	-24052.163
**241.567**	204.548	24838.4	-24573.647
**244.148**	206.559	25326.9	-25104.067
**246.746**	208.591	25825.5	-25643.495
**249.367**	210.636	26332.9	-26192.767

**250.682**	211.652	26587.6	-26469.747
**252.659**	213.192	26974.9	-26889.978
**254.645**	214.736	27366.6	-27314.849
**256.64**	216.288	27763.4	-27744.752
**258.643**	217.848	28165.3	-28179.56
**260.655**	219.418	28572.9	-28619.499
**262.673**	221.011	28989.8	-29063.822
**264.691**	222.681	29430.3	-29511.357
**266.702**	224.485	29909.4	-29961.198
**268.629**	227.029	30590.6	-30395.973
**270.643**	228.908	31097	-30855.348
**272.705**	230.526	31536.6	-31328.993
**274.775**	232.158	31983.3	-31807.914
**276.856**	233.781	32431	-32292.673
**278.731**	235.267	32844	-32732.206
**280.841**	236.934	33310.3	-33230.481
**282.967**	238.607	33782	-33735.907
**285.113**	240.302	34263.5	-34249.724
**287.278**	242.016	34753.8	-34772.072
**289.4**	243.698	35238.8	-35287.401
**292.211**	245.929	35887.8	-35975.359
**295.047**	248.187	36550.7	-36676.13
**297.909**	250.469	37227.2	-37389.769
**300.796**	252.767	37915.1	-38116.203
**303.708**	255.088	38616.7	-38855.566
**306.645**	257.44	39334.3	-39608.389
**309.605**	259.818	40067.4	-40373.552
**312.59**	262.225	40815.9	-41153.013
**315.599**	264.659	41580.4	-41945.716
**318.633**	267.118	42360.3	-42752.31
**321.691**	269.6	43155.1	-43572.794
**324.772**	272.106	43964.9	-44407.51
**327.878**	274.635	44790.2	-45256.575
**331.4**	277.507	45737	-46228.82
**332.804**	278.654	46119.1	-46618.066
**335.622**	280.961	46889.9	-47406.793
**338.45**	283.282	47672.2	-48204.593
**341.287**	285.621	48467.1	-49011.634
**344.129**	287.99	49279.1	-49826.611
**346.974**	290.411	50115.7	-50649.366
**349.817**	292.923	50990.9	-51478.545
**352.642**	295.618	51937.3	-52310.023

**355.411**	298.733	53040	-53132.994
**358.018**	302.949	54544.1	-53917.095
**360.15**	310.245	57164.1	-54570.637
**361.356**	323.476	61937	-54952.993
**361.866**	341.18	68339.2	-55122.242
**362.189**	360.274	75251.7	-55235.58
**364.594**	366.179	77397.3	-56109.366
**367.52**	368.87	78382.5	-57184.602
**370.458**	371.552	79372.3	-58272.111

[a] Data obtained by extrapolation, metastable form H and G relative to stable form in Table XS2. S ≠ 0 at 0 K may be due to experimental error or the extrapolation of Cp to 0K.

CLAUSIUS-CLAPEYRON EQUATION AND THE VAPOUR PRESSURES OF THE CONDENSED PHASES

The Clausius-Clapeyron equation can be written as:

$$\ln P = -\frac{\Delta H}{RT} + B \qquad \text{(S3)}$$

In this equation *P* is the vapour pressure of the condensed phase, Δ*H* is the enthalpy of vaporization in case of the liquid and the sublimation enthalpy in case of a solid, *R* is the gas constant, 8.314472 J K^{-1} mol^{-1}, *T* is the temperature in kelvin and *B* is a constant.
Using the boiling point and the enthalpy of vaporization of 583.85 K and 55160 kJ mol^{-1} obtained from ACD/Labs (ACDLabs *Advanced Chemistry Development (ACD/Labs) Software*, V11.02), the constant *B*vap can be calculated. It should be realised that at the boiling point the vapour pressure of the liquid is equal to the pressure in the atmosphere or 1×10^5 Pa. Since Δ*H*, *R*, and *T* are known in eq. S3, *B* can be obtained. The results can be found in Table S8. With these parameters the vapour pressure of the liquid can be calculated at the melting point of form II and because the liquid and form II are at equilibrium at the melting point, their vapour pressures must be the same (triple point II-*l*-*v*). Moreover, the enthalpy of vaporization is known, thus through thermodynamic cycling, the enthalpy of sublimation of form II should be the enthalpy of vaporization with the melting enthalpy added to it: $\Delta_{II\to v}H = \Delta_{l\to v}H + \Delta_{II\to l}H$ = 55160 + 22038 = 77198 J mol^{-1}. With this information B_{II} can be calculated. The same can be done for form I, using as enthalpy of sublimation $\Delta_{I\to v}H = \Delta_{l\to v}H + \Delta_{II\to l}H + \Delta_{I\to II}H$ = 77828 J mol^{-1}. The pressure at the fusion of form I can be calculated with the equation for the liquid leading to 83 Pa and with this information the constant B_I can be determined. In the same way the vapour pressure of form III can be obtained.

Table S8. Vapour pressure calculations [a]

Phase	Transition	Temperature /K	Pressure /Pa	ΔH /J mol^{-1}	B
**Liquid (*l*)**	boiling	583.85	1×10^5	55160	*22.88*
**II**	fusion II	362.0	*95*	77198	*30.20*
**I**	fusion I	359.5	*83*	77828	*30.46*
**III**	fusion III	358.3	*78*	77648	*30.42*

[a] italic values have been calculated using eq. S3 and R = 8.314472 J K^{-1} mol^{-1}

Once the vapour pressure data of each condensed phase is given by the Clausius-Clapeyron equation (eq. S3), it can be demonstrated which phase is the most stable by comparing the vapour pressures reflecting the vapour pressure equilibrium lines in Figure S2 and Figure 9. The phases in equilibrium will have the same vapour pressure, whether they are the most stable phases or not. In the case of the I-III-*v* triple point, because its temperature is very high, the vapour pressure for the solid phases is in the order of MPa. The most stable phase may appear to be the liquid phase with 0.3 MPa, however, it should not be forgotten that atmospheric pressure is 0.1 MPa, which implies that even the liquid is not stable anymore at this temperature. The pressures at the other triple points involving the vapour phase are very low in terms of MPa. The most stable phase always has the lowest vapour pressure.

Table S9. Vapour pressures of individual phases at triple points [a]

Triple point	P_I /Pa	P_{II} /Pa	P_{III} /Pa	P_l /Pa
**II-*l*-*v* (1)**	100	95	102	95
**I-*l*-*v* (2)**	83	79	86	83
**III-*l*-*v* (3)**	76	73	78	78
**I-II-*v* (7)**	2.4	2.4	2.5	6.8
**III-II-*v* (8)**	0.011	0.012	0.012	0.15
**I-III-*v***	7.4×10^6	6.4×10^6	7.4×10^6	0.3×10^6

[a] The numbers in parentheses correspond to the triple points in Figure S2 below.

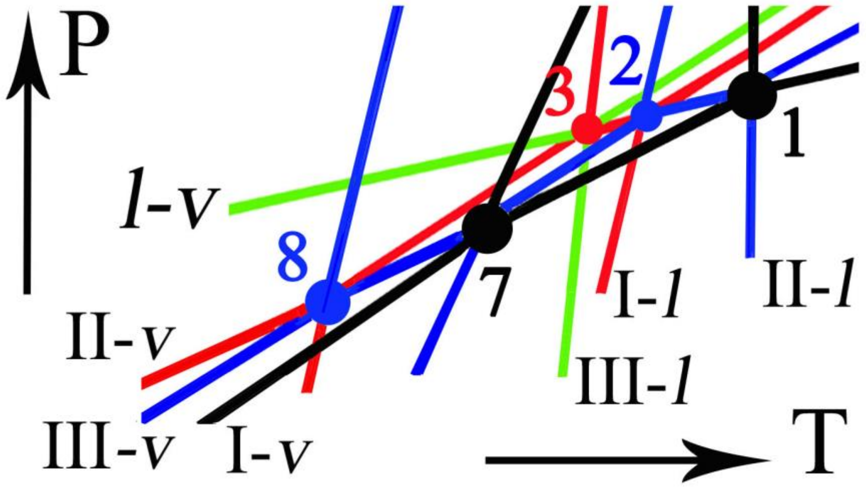

Figure S2. Schematic close-up of the melting and vapour equilibria in the pressure temperature phase diagram of benzocaine including the vapour equilibria. Solid black line, black circle: stable, blue line/circle: metastable, red line/circle: supermetastable, green line: hypermetastable.
